# Context-aware heatstroke relief station placement and route optimization for large outdoor events

**DOI:** 10.1186/s12942-021-00275-z

**Published:** 2021-05-25

**Authors:** Yan Wu, Tianqi Xia, Adam Jatowt, Haoran Zhang, Xiao Feng, Ryosuke Shibasaki, Kyoung-Sook Kim

**Affiliations:** 1 The University of Tokyo, Kashiwanoha 5-1-5, Kashiwa, 277-0882 Japan; 2 Xi’an Jiaotong University, 28 Xianning West Road, Xi’an, 710049 Shaanxi China; 3 National Institute of Advanced Industrial Science and Technology, Aomi, Koto, Tokyo, 135-0064 Japan; 4 University of Innsbruck, Innrain 52, 6020 Innsbruck, Austria

**Keywords:** Optimization, Context-aware, Pedestrian flow, Olympic games

## Abstract

**Background:**

Heatstroke is becoming an increasingly serious threat to outdoor activities, especially, at the time of large events organized during summer, including the Olympic Games or various types of happenings in amusement parks like Disneyland or other popular venues. The risk of heatstroke is naturally affected by a high temperature, but it is also dependent on various other contextual factors such as the presence of shaded areas along traveling routes or the distribution of relief stations. The purpose of the study is to develop a method to reduce the heatstroke risk of pedestrians for large outdoor events by optimizing relief station placement, volume scheduling and route.

**Results:**

Our experiments conducted on the planned site of the Tokyo Olympics and simulated during the two weeks of the Olympics schedule indicate that planning routes and setting relief stations with our proposed optimization model could effectively reduce heatstroke risk. Besides, the results show that supply volume scheduling optimization can further reduce the risk of heatstroke. The route with the shortest length may not be the route with the least risk, relief station and physical environment need to be considered and the proposed method can balance these factors.

**Conclusions:**

This study proposed a novel emergency service problem that can be applied in large outdoor event scenarios with multiple walking flows. To solve the problem, an effective method is developed and evaluates the heatstroke risk in outdoor space by utilizing context-aware indicators which are determined by large and heterogeneous data including facilities, road networks and street view images. We propose a Mixed Integer Nonlinear Programming model for optimizing routes of pedestrians, determining the location of relief stations and the supply volume in each relief station. The proposed method can help organizers better prepare for the event and pedestrians participate in the event more safely.

## Background

With global warming and heat island effects caused by urbanization, heatstroke is becoming an increasingly severe threat to outdoor activities in summer [1, 2]. On the other hand, summer is a popular season for outdoor trips due to summer vacations and many events that normally take place in this season. In such a case, it is fundamental to reduce heatstroke risk when holding large outside events, especially for pedestrians who are likely to be exposed to high temperatures [3].

There are several strategies that local governments or event holders could take to reduce heatstroke during large outdoor events. The easiest one is to set outdoor events to an earlier or later time of the day [4]. However, this strategy would potentially reduce the number of visitors for early events, or increase the cost and other risks for nighttime events. Considering the potential income loss and cost increase, it is usually more reasonable for event organizers to minimize the risk in other ways such as providing relief stations to give first-aids to those who suffer or may suffer from heatstroke, as well as carefully planning routes that pose the smallest risk [5]. However, simply implementing either of these two strategies could be rather challenging when the walkable space is complicated, e.g., spanning a large area with multiple origins and destinations (OD) and with complex routes between many OD pairs. In such a case, setting relief stations for all the routes would translate to a high cost. On the other hand, though heatstroke risk could be reduced by enforcing visitors to choose the optimal routes predefined by the organizers, heatstroke risk on long routes could be still inevitably high. To prevent these scenarios, setting up temporal relief stations based on predefined routes would ensure safer travel and at the same time keep the cost under control.

On the other hand, the location optimization scenario of heatstroke prevention varies from other similar scenarios in that heatstroke risk is sensitive to different environmental contexts within the walkable space [6]. Specifically, a road segment with a better physical environment (e.g., an environment with less solar radiation) might pose less heatstroke risk than the road segment with a poor physical environment (e.g., an environment with more solar radiation). As a result, pedestrians walking along the routes with high heatstroke risk cause high potential demand for assistance such as providing shelter or water. However, the traditional data used for representing spatial contexts, such as public satellite images and statistic information, are usually characterized by low spatial or temporal resolution that cannot be applied to represent the detailed environmental context [7, 8]. As a result, the existing studies usually have the Uncertain Geographic Context Problem (UGCoP) [9] of ignoring health risk variance in different spatial and temporal units.

The development of big data and location-based services (LBS) makes however detailed contextual information available for reasoning about the health risk of diverse locations. Different from the traditional data collection and processing approaches, contextual information can be now collected through road network-based sensors such as cars and street cameras. The data provided thanks to the deployment of such sensors can help to distinguish contextual differences in road networks when it comes to health risks such as heatstroke. Based on large contextual data, our study proposes a problem of optimizing routes and placement of heatstroke relief stations in a road network within walkable spaces. The objective of this problem is to minimize global heatstroke risk, which is calculated by microscope contextual information on each road segment. Specifically, we propose a heatstroke risk model that measures the heatstroke risk of each road segment with different indicators calculated using heterogeneous data including characteristics of road segments. Based on this model, we conduct a case study in a real-world scenario of the Tokyo Olympic Games with heterogeneous data collected from different data sources including Olympic schedules, facility locations and road network information. With the calculated contextual heatstroke risk, we further propose a Mixed Integer Nonlinear Programming (MINLP) model to optimize pedestrian routes, relief station locations and supply volume of each station at different times.

The contributions of this study can be listed as follows:

1. We propose a novel emergency service problem that can be applied in large outdoor event scenarios with multiple walking flows.
2. We introduce a dedicated framework to solve this task. Both supply and demand are considered during the facility optimization. Specifically, the demand in this study is represented by pedestrian flows instead of demand points.
3. The model we propose does not only plan the placement of relief stations and supply volume scheduling but also determines the optimal routes for the dynamic pedestrian flows.

## Related work

### Emergency facility location optimization

Emergency facilities are of great significance in public health as they can provide first aid to emergency victims to reduce casualties. Comparing to non-emergency facilities, the demand for emergency facilities is more time-sensitive and dependent on particular emergency scenarios. Therefore, although the existing studies on emergency facility location optimization (EFLO) have similar objectives with other FLO problems to maximize covering location (MCLP) [10] or minimize cost or the number of facilities while making sure that the entire target region remains covered [11], the existing studies distinguish themselves from other EFLO problems in terms of optimization targets and problem settings [12].

From the perspective of optimization targets, several types of emergency facilities have received attention in existing studies. Off-site public access devices (OPAD) usually refer to those facilities that provide medical service out of regular healthcare facilities, e.g., automated external defibrillator (AED). Siddiq et al. [13] pointed out that the limited accessibility, poor visibility and lack of registration could influence AED demand and set different coverage values for different devices in location optimization.

The emergency center or department is another type of common emergency facility. Different from the OPAD devices, each emergency center usually has a larger supply volume and higher cost. Thus, in addition to studies of setting permanent emergency centers or departments with coverage problem settings [14], a lot of studies have been conducted on optimizing locations for temporary relief emergency centers. In these studies, emergency medical service demand, supply and accessibility distribution is supposed to vary in different emergency scenarios and their phases [15]. Schempp et al. [16] proposed a framework of utilizing social networking services (SNS) data to detect the emergency demand distribution and optimize the temporal rescue centers via global particle swarm optimization and mixed-Integer linear programming. Oran et al. [17] proposed a location-routing problem that considers the propriety of the locations and solves the problem using an mix integer programming solver.

Finally, ambulance transportation is significant in emergency medical services (EMS) and has received much attention in some existing studies. Comparing to other emergency facilities, the optimization targets of ambulance transportation are not limited to the location of ambulance stations, but also include the relocation and dispatching of ambulances. Since the supply volume of an ambulance fleet is limited, a problem that can represent the vacancy of ambulances is necessary for real-world application. This problem could be either solved by deterministic models via the backup of multiple ambulances to cover EMS demands [14], or solved by the probabilistic models that represent the access information by the probability of ambulance vacancy [18]. Daskin [19] proposed a maximum expected coverage location problem (MEXCLP) that the expected coverage of ambulances is calculated by the vacant probability. The MEXCLP could be improved via short-term dynamic settings of ambulance supply and demand [20].

The problem approached in this study distinguishes itself from the existing EFLO problems in the following aspects: first, we assume the potential "patients" of our problem are pedestrians; second, the relief stations are not the destinations of the pedestrians, and the heatstroke risk is generated during the trip. Altogether these differences make our task a novel research problem in the field of emergency service.

### Context-aware LBS application

Context-aware LBS application refers to those LBS applications that can provide service based on their present context including location, time and companions [21]. This extra information is of great significance to application users as their contexts vary from time to time, and any analysis with uncertain contextual information will generate bias and reduce application utility [9].

The last decade has witnessed the great development of contextual-aware LBS applications due to the availability of spatial and temporal data in high resolution [22]. A common context-aware implementation in the LBS application is to recommend points of interest (POI) to the visitors based on their spatio-temporal information, profiles and historical records. Yao et al. [23] proposed a tensor-factorization-based recommender system to recommend POIs with multi-dimensional contextual information. Besides single POI, several studies focused on recommending POI sequences. Chen and Jiang [24] proposed a context-aware personalized POI sequence recommendation system to recommend a sequence of POIs via reinforcement learning. Laß et al. [25] represented POIs by a graph and incorporated contextual information including historical records and traveling time into the traditional two-dimension user-item recommender system.

Another important implementation of context-aware information in LBS applications is route recommendation and navigation. In routing applications, contextual information could be utilized to measure the quality of each road segment to improve the traditional routing application by providing users with scenic, safe or attractive routes among other dimensions [26]. Specifically, the contextual information is utilized to evaluate each road segment based on its attractiveness or risk and choose the roads which are more attractive or less dangerous for different application scenarios. Attractiveness is usually evaluated by the accessibility to POIs [27] or the landscape diversity [28] while risk can be assessed by social environment represented by metrics such as accidents, crimes and population density[29, 30], or physical environment such as solar radiation and infrastructure preparedness [31]. Generally, the contextual information could be collected from official statistical data [32], location-based social networking (LBSN) platforms [29], or web map services [33]. Unlike the above-mentioned researches focused on recommending individual optimal routes, this study focuses on recommending routes for a group of people with the global optimal objective.

## Methods

### Problem definition and setting

Let us assume there is a planned large, long-lasting event that consists of several sub-events to be held in a given area. The area is composed of a road network with multiple venues, hotels, train stations and scenic spots that will be origin/destinations (ODs) for walking users. At different periods of each day, different sub-events will be held in different venues. During walking outdoors between ODs, pedestrians are at risk of heatstroke. In this study, many factors that affect the heatstroke risk of pedestrians, such as walking distance, solar radiation, pedestrian flow (which is represented simply by ‘flow’ in the following) density, the number and location of relief stations and the supply volume, are taken into the consideration.

The proposed problem is set as optimizing the number, location and supply volume of each temporary station as well as the pedestrian routes in the road network to reduce heatstroke each day during the large outdoor event. It should be emphasized that the solution of the problem corresponds to the scheduling scheme for all the sub-events every single day during the event. Before the optimization, there are some preparatory works needed to be done: (a) facilities and POIs extraction; (b) extraction and simplification of the road network for a given area; (c) the extraction and calculation of the heatstroke related data; (d) event schedule collection and pedestrian flow simulation. By handling the preparatory work and solving the optimization problem, we are not only able to provide event holders with a reasonable allocation scheme of relief stations and supply volume but also to recommend walking paths for pedestrians.

The optimization setting in our research can be described as follows:

Assumption:

1. There will be several inflows before each event and outflows after the event between event venues and other facilities such as places of interests (POIs), hotels and stations.
2. The location of the relief station cannot be changed during the day, the supply volume can be however reassigned at different times of the day.

Input:

1. The road network information including nodes, edges, the length of each edge, and the factor value that increases the vulnerability of each edge, the coordinates of each node.
2. The Environment related data of the given area.
3. The numbers of time units and simulated flow density at each time interval.
4. The sets of start nodes and end nodes of all flows.
5. The maximum number of relief stations and supply volumes of each station, the maximum allowed heatstroke risk of all edges (road) in the road network, the edge set on the path of each flow and the edge set between every two adjacent stations on the path of each flow.

Determine:

1. The numbers and locations of relief stations on a given day.
2. The supply volume of each relief station at different time units.
3. The optimal route is made up of a set of road segments with the least heatstroke risk of different flows at different time units.

To mathematically model the problem, we represent the road network as a graph $G = \left( {V,E} \right)$ that consists of the vertex set *V* and edge set *E*. Specifically, a vertex $v \in V$ of the road network represents either an origin or destination point of flows or the intersections of the road segments. Then we propose a heatstroke risk metric to measure the risk of each road segment at different times during the events with different indicators including both pre-calculated parameters and the decision variables to be optimized. With the objective function of minimizing global risk values, a Mixed Integer Nonlinear Programming (MINLP) model [34] is established to work out the optimal solutions. MINLP model refers to a model whose decision variables include integer variables and continuous variables and, at the same time, the objective function or constraint condition contains the nonlinear form of the decision variable. For this type of model is difficult to guarantee the global optimal solution. However, the relatively optimal solution can be obtained by using a meta-heuristic algorithm such as genetic algorithm. All notations of the mathematical models that we are going to introduce are listed and described in the section of Nomenclature.

### Measuring heatstroke risk

We use a framework of a traditional risk model with heterogeneous data collected from different data sources. A traditional risk model divides risk into three factors which are hazard, vulnerability and exposure. Then a simple approach for measuring emergency risk is realized by multiplication of these three factors:$R = r_{hazard} \times r_{vulnerability} \times r_{exposurre}$.Generally, the hazard represents the possibility that the emergency happens [35] while vulnerability represents the lack of proper resistance to the emergency, which is dependent on the context information. Finally, exposure refers to the amount of time spent when exposed to the hazard or the number of people involved. Although several studies have been applied to estimate the heatstroke risk on a macro scale [36], few focus on microanalysis, which should have different indicators depending on the distinct micro context. We utilize different micro indicators to implement our risk model for micro heatstroke analysis. The indicators of the hazard, vulnerability and exposure factors are listed as follows:

#### Hazard

Hazard is measured by Wet Bulb Globe Temperature (WBGT) which has been applied in other heatstroke-related studies [36, 37]. Specifically, we choose a data-driven approach to measure heatstroke hazard via historical WBGT data at different hours during summertime. We utilize a normalized index *W*_*t*_ to represent the probability of the heatstroke severity for each hour *t*. In particular, for each hour we evaluate the average WBGT in all summer days. Then the average WBGT is normalized by the min and max temperature based on the government guidance.^1^

#### Vulnerability

Vulnerability is the factor related to the contextual environment. Generally, the vulnerability could be generated by the existing contextual environment, or reduced by improving the environment via temporary service. In this study, vulnerability is denoted by the indicators of a road segment. Sky view factor (SVF) defines the ratio of sky hemisphere visible from the ground that is not obstructed by buildings, terrain or trees [38]. SVF has been proved to be quite an important indicator for computing solar radiation related to heatstroke. Generally, higher SVF in a place denotes more solar radiation, making the place more vulnerable to heatstroke [39]. Therefore, in our model SVF is utilized to measure the vulnerability of the existing contextual environment. On the other hand, the relief stations are set to reduce vulnerability and a station with a larger service volume (e.g., more volunteers) could help more pedestrians. Therefore, Vulnerability $R_{i,t}^{V}$ could be measured by Eq. (1) with a given SVF value $V_{i}^{I}$ and the vulnerability reduction indicator $V_{i,t}^{R}$ computed from the supply volume $N_{i,t}^{V}$ in each road segment *i* and time interval *t*.

1 $$R_{i,t}^{V} = \frac{{V_{i}^{I} }}{{V_{i,t}^{R} }} = \frac{{V_{i}^{I} }}{{1 + \left( {B_{i}^{S} N_{i,t}^{V} } \right)^{d} }} \, \forall i \in E,t \in T$$

where $B_{i}^{S}$ are binary variables that refer to whether there is a relief station on the road segment *i*,$N_{i,t}^{V}$ are integer variables that refer to the volumes of all the relief stations on the road segment *i* at time interval *t*. Finally, *d* is an index of vulnerability reduction indicator and is set to 1 in this research.

#### Exposure

Exposure is measured by the total walking time of all pedestrians for each road segment. To simplify the calculation, we assume all pedestrians walking at the same speed in all road segments and within all-time intervals. As a result, the exposure of heatstroke in this study is proportional to the number of pedestrians and the road length for each road segment. Therefore, for a given road segment *i* at time interval *t*, exposure volume is denoted by Eq. (2)

2 $$R_{i,t}^{E} = L_{i} \sum\limits_{f} {B_{i,f,t}^{P} N_{f,t}^{P} } \, \forall i \in E,f \in F,t \in T$$

where $L_{i}$ is the length of road segment *i*,$B_{i,f,t}^{P}$ are binary variables referring to whether flow *f* is observed on edge *i* at time interval *t*, while $N_{f,t}^{P}$ denotes the number of people of flow *f* at time interval *t*.

With the factors defined, the risk for flow *f* in road segment *i* at time interval *t* could be denoted by Eq. (3).

3 $$\begin{gathered} R_{i,f,t} = B_{i,f,t}^{P} \left( {W_{t} } \right)^{a} \left( {R_{i,t}^{V} } \right)^{b} \left( {R_{i,t}^{E} } \right)^{c} \hfill \\ \, = B_{i,f,t}^{P} \left( {W_{t} } \right)^{a} \left( {\frac{{V_{i}^{I} }}{{1 + \left( {B_{i}^{S} N_{i,t}^{V} } \right)^{d} }}} \right)^{b} \left( {L_{i} \sum\limits_{f} {B_{i,f,t}^{P} N_{f,t}^{P} } } \right)^{c} \, \forall i \in E,f \in F,t \in T \hfill \\ \end{gathered}$$

where $W_{t}$ is the hazard factor defined by WBGT score during the time interval *t*.

### Optimization model

With the risk metric defined above, this study provides an MINLP model to work out the solutions for facility and path optimization with one objective function and several constraints. The model is solved by genetic algorithm with several strategies to accelerate the computation process.

#### Objective function

The objective function is to minimize the total heatstroke risk, i.e., the risk value generated by the risk metric introduced in the last section, for all flows during all the events within a time interval *T*, which can be denoted by Eq. (4):

4 $$\min \, Risk = \sum\limits_{i} {\sum\limits_{f} {\sum\limits_{t} {R_{i,f,t} } } } \quad \forall i \in E,f \in F,t \in T$$

#### Constraints

In this study, several constraints are set either for solutions to meet the predefined parameters or for overcoming the shortages of a single objective function for enabling more practical application. Generally, constraints can be categorized into the following four groups based on their target:

A. Station constraints

The total number of supply stations established cannot exceed the maximum. It is described as below:

5 $$B_{i}^{S} = \left\{ \begin{gathered} 1, \, if \, \exists i = E_{s}^{S} \hfill \\ 0, \, if \, \forall i \ne E_{s}^{S} \hfill \\ \end{gathered} \right. \, \forall i \in E,s \in S$$

6 $$\sum\limits_{i} {B_{i}^{S} } \le N^{S,max} \, \forall i \in E$$

B. Flow path constraints

In this study, since the flows are represented by a set of edges from the given origin and destination nodes, the path constraints mainly focus on edge connectivity and origin–destination connectivity.

Specifically, the origin–destination connectivity constraint denotes that the start (end) edge should be the only edge connected to the origin (destination) node. These constraints are as follows:

7 $$\sum\limits_{i} {B_{i,f,t}^{P} } = 1 \, \forall i \in E^{SE} ,f \in F,t \in T$$

8 $$\sum\limits_{i} {B_{i,f,t}^{P} } = 1 \, \forall i \in E^{EE} ,f \in F,t \in T$$

For each flow path at time interval *t*, the connectivity is judged by two Boolean matrices with the size of $n_{f,t}^{E}$, adjacency matrix $A_{f,t}$ and the reachability matrix $P_{f,t}$ of the selected edges. Adjacency is represented by Constraint (11) which means if there is a point connected by both edge *i* and edge *j*, then the two edges are adjacent, the value of $a_{i,j,f,t}$ is 1, otherwise it is 0. Reachability matrix $P_{f,t}$ is obtained by Boolean addition and Boolean multiplication for adjacency matrix which are described by Eq. (13) and Eq. (14), and constraint (15) ensures that the graph composed of the selected edges is connected.

9 $$n_{f,t}^{E} = \sum\limits_{i} {B_{i,f,t}^{P} } \quad \forall i \in E,\,f \in F,\,t \in T$$

10 $$A_{f,t} = \left( {a_{i,j,f,t} } \right)_{{n_{f,t}^{E} \times n_{f,t}^{E} }} \, \forall i,j \in E^{FP} ,f \in F,t \in T$$

11 $$a_{i,j,f,t} = \left\{ \begin{gathered} 1, \, if \, N_{{_{i,f,t} }}^{EF} = N_{{_{j,f,t} }}^{EF} \, or \, N_{{_{i,f,t} }}^{EF} = N_{{_{j,f,t} }}^{ES} \, or \hfill \\ \, N_{{_{i,f,t} }}^{ES} = N_{{_{j,f,t} }}^{EF} \, or \, N_{{_{i,f,t} }}^{ES} = N_{{_{j,f,t} }}^{ES} \, \hfill \\ 0, \, otherwise \hfill \\ \end{gathered} \right. \, \forall i,j \in E^{FP} ,f \in F,t \in T$$

12 $$P_{f,t} = \left( {p_{i,j,f,t} } \right)_{{n_{f,t}^{E} \times n_{f,t}^{E} }} \, \forall i,j \in E^{FP} ,f \in F,t \in T$$

13 $$P_{f,t} = \bigcup\limits_{k = 1}^{{n_{f,t}^{E} }} {A_{f,t}^{\left( k \right)} } \, \forall f \in F,t \in T$$

14 $$A_{f,t}^{\left( k \right)} = A_{f,t}^{{\left( {k - 1} \right)}} \odot A_{f,t} \, \forall f \in F,t \in T$$

15 $$p_{i,j,f,t} = 1 \quad \forall i,j \in E^{FP} ,f \in F,\,t \in T$$

C. Volume constraints

The following volume constraints ensure that the total volume of service number of all supply stations at any time interval cannot exceed the max value. In addition, the constraint that the volume number of supply stations on each road segment should not exceed the maximum function is realized by implementing a sufficiently large constant *M*.

16 $$N_{i,t}^{V} = \left\{ \begin{gathered} \sum\limits_{s} {N_{s,t}^{V} \, } , \, if \, i = E_{s}^{S} \hfill \\ 0, \, otherwise \hfill \\ \end{gathered} \right. \, \forall i \in E,s \in S,t \in T$$

17 $$\sum\limits_{i} {\left( {B_{i}^{S} N_{i,t}^{V} } \right)} \le N^{V,max} \, \forall i \in E,t \in T$$

18 $$N_{i,t}^{V} \ge M\left( {B_{i}^{S} - 1} \right) + 1 \, \forall i \in E,t \in T$$

19 $$N_{i,t}^{V} \le M\left( {1 - B_{i}^{S} } \right) + N^{SV,max} \, \forall i \in E,t \in T$$

where $N_{s,t}^{V}$ is the volume of relief station *s* at time interval *t*.

D. Risk constraints

Risk constraints are set to exclude those solutions with concentrated stations in adjacent road segments. Specifically, the constraints should ensure that the value of the risks at each edge ($R_{i,t}$), at all edges of each flow ($\sum\limits_{i} {R_{i,f,t} }$) and between every two adjacent stations on the path of flow *f* ($\, R_{s,k,f,t}$) should not exceed the predetermined max risk values. The constraints are listed as follows:

20 $$R_{i,t} \le Ri^{max} \quad \forall i \in E,\,t \in T$$

21 $$\sum\limits_{i} {R_{i,f,t} \le Rf^{max} } \quad \forall i \in E,\,f \in F,\,t \in T$$

22 $$\, R_{s,k,f,t} \le Rs^{max} \quad \forall s,k \in S,\,f \in F,\,t \in T$$

### Optimization algorithm

The proposed MINLP problem is NP-hard and it is time-consuming to directly apply any solution to the proposed model due to a large number of variables. We then apply several strategies to effectively generate efficient solutions.

In particular, the problem is solved by genetic algorithm (GA). GA is a widely used effective algorithm with good performance of global search and strong robustness. It is suitable for solving complex optimization problems that can be described as mixed linear models or mixed nonlinear models. The GA algorithm in this work starts with a set of the initial population that is generated based on certain requirements rather than randomly generated. This is beneficial to improve the convergence rate and the quality of the solution. Then the algorithm evaluates each individual in the population through the fitness function. A certain proportion of individuals at the top are selected as elite individuals and directly retained in the next-generation population. The next-generation population also includes children who are reproduced by selected elite individuals through crossover and mutation. With the process of evolution, the solution gradually approaches the optimal solution according to the principle of the survival of the fittest.

#### Generating initial GA population

Inspired by the assignment of initial solutions to ant colony optimization (ACO) in [40], the proposed method generated an initial GA population with feasible solutions to accelerate the convergence. In this study, we choose a group of paths based on the actual situation of excluding the solutions with large detours. Specifically, we apply Dijkstra algorithm to generate the shortest paths with the least SVF weighted length. Dijkstra is a classic algorithm for finding the shortest path from a given starting vertex *x* to all *n*-1 other vertices in a positively weighted graph. Then a depth-first search is applied to acquire all paths with the edge number smaller than or equal to the edge number in the shortest path + 5. Then the initial population can be represented by a combination of different paths for different flows.

#### Penalty function

To get solutions that satisfy constraints in the presented model, we use a penalty function to exclude the chromosomes that cannot meet the constraints in the evolution. Then the fitness function by which the next generation is bred could be represented by a sum of the objective function and penalty function, which is shown as Eq. (23).

23 $$\begin{aligned} F\left( {{\mathbf{X}},\delta_{n} } \right) & = f\left( {\text{X}} \right) + \delta_{1} \sum\limits_{i = 1}^{{n^{p} }} {\left( {p_{i} \left( {\mathbf{X}} \right) - 1} \right)} + \delta_{2} \sum\limits_{j = 1}^{{n^{v} }} {\max \left\{ {0,\left( {v_{i} \left( {\mathbf{X}} \right) - N^{V,max} } \right)} \right\}} + \delta_{3} \sum\limits_{k = 1}^{{n^{ri} }} {\max \left\{ {0,\left( {ri_{i} \left( {\mathbf{X}} \right) - Ri^{max} } \right)} \right\}} \hfill \\ & \quad + \delta_{4} \sum\limits_{p = 1}^{{n^{rf} }} {\max \left\{ {0,\left( {rf_{i} \left( {\mathbf{X}} \right) - Rf^{max} } \right)} \right\}} + \delta_{5} \sum\limits_{q = 1}^{{n^{rs} }} {\max \left\{ {0,\left( {rs_{i} \left( {\mathbf{X}} \right) - Rs^{max} } \right)} \right\}} \hfill \\ \end{aligned}$$

where ${\mathbf{X}}$ represents a chromosome, i.e., a potential solution to the problem.$f\left( {\text{X}} \right)$ is the objective function while the remaining items are penalty functions.$\delta_{k} \left( {k = 1,2,3,4,5} \right)$ represents the weight of each penalty function which is usually a sufficiently large constant. The more variables that do not meet the constraints, the greater the value of the penalty function. Besides, the objective function is to minimize the risk of heatstroke. Therefore, the smaller the value of fitness, the better the solution. In addition, to further speed up the convergence, we compute the fitness function of the entire population in parallel.

## Case study: an application scenario for Tokyo Olympic Games in Tokyo Waterfront City

### Study area

**Fig. 1:**
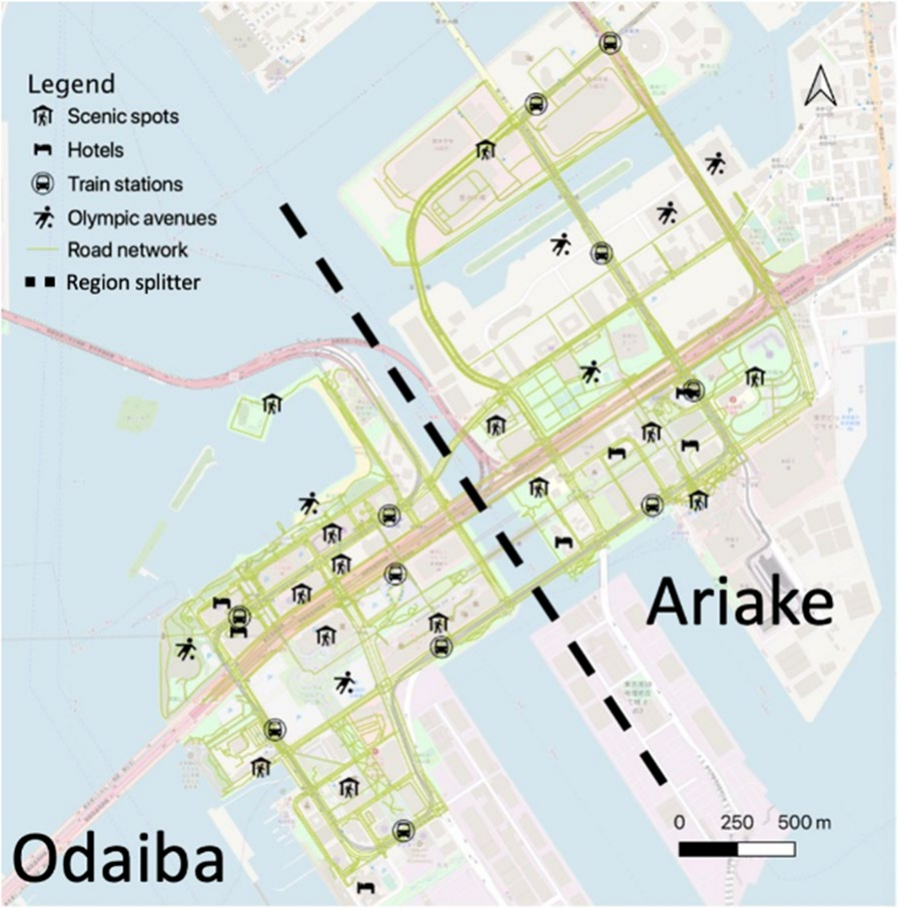
Spatial distribution of POIs in TWC

Based on the model proposed above we conduct experiments on the walkable space of Olympic venues in the Tokyo Waterfront City (TWC) which mainly includes the regions of Odaiba (Aomi included) and Ariake. During the Tokyo Olympic Games, a lot of games are scheduled to be held in TWC. Besides, as a region with concentrated scenic spots, shopping malls and theme parks, TWC attracts a lot of visitors every year and ranks 12th among 4,027 scenic spots in the central Tokyo area.^2^ The abundant scenic spots and hotels distributed in TWC make it a space with the forecasted high demand for walking during the Olympic Games as there will be a lot of visitors walking to the scenic spots near Olympic venues[41]. Figure 1 shows the map of the two main regions in TWC with different types of facilities and the extracted road network.

### Data collection and preprocessing

#### POI and facility extraction

We collect different types of POIs including scenic spots, hotels, railway stations and Olympic venues from heterogeneous data sources. Scenic spots and hotel data are taken from TripAdvisor, railway stations from the National Land Numerical Information and avenue locations are taken from their official websites. Starting with the initial number of 400 raw POIs in Odaiba area collected from the TripAdvisor, we next merge them based on their spatial entities to remove duplicated POIs in the same building and we use the total comment numbers in each location as its popularity.

#### Road network

Road network is collected from OpenStreetMap (OSM) with the help of the library Osmnx [42]. In TWC region, the raw data collected from OSM include more than 4,000 road links including different road types, which makes it difficult to be directly applied to the optimization problem for pedestrians. Thus, we simplify the road network based on the extracted skeleton [43, 44] and attributes of roads: levels and types. After this simplification, the total number of road segments in TWC area is reduced to 234, in which 131 segments are located in Odaiba and 103 segments are located in Ariake.

#### Heatstroke related data collection and processing

A. WBGT data

WBGT data is collected from the government website^3^ at one-hour intervals. To represent the situation during the Tokyo Olympic Games, we take data on the same day from 2017 to 2019 to calculate the normalized index.

B. SVF data

In order to calculate SVF for each road segment, we refer to the work of [44] to collect Google Street View images for each simplified road segment in the TWC area and we conduct image segmentation to extract the sky range in the images. Specifically, we generate intermediate points on each road segment with a five-meter interval, then use the coordinates of the points and nodes as the request parameters for Google API to gather panorama data. Having collected the panorama data, we utilize a SegNet model [45] trained by CityScape dataset [46] for image detection and convert the detection results to fisheye images to calculate SVF values.

#### Event schedule collection and flow simulation

In the case study, events represent the sports held in the venues of TWC during the Olympic Games. For each sport event, the official schedule of time, location^4^ and the estimated audience number^5^ are collected from the official website. In total there will be 71 sports events held during 16 days in the whole research area.

To simplify the computation, we choose in this study an hour as the time unit for calculating the flows. For each event, its estimated audience number is distributed as the total inflows within two-time units (two hours) just before the event. Similarly, the audience number is taken to represent the total outflows distributed during the two-time units (two hours) right after the event. Since there are no records for allocating the total flow volumes to individual flows, we apply the Huff model [47] to simulate the flow number between venues and other facilities which takes both distance decay and the facility popularity into consideration.

### Experiments and results

Having the data collected and processed as explained above, we conduct in this case study several experiments with different data inputs for solving the optimization problem under different flow numbers and contextual information.

On one hand, TWC has an area of 400 ha which is too large to form a single walkable space and the distance of facilities between its two regions (Odaiba and Ariake) is relatively far (as shown in Fig. 1; it is necessary to go across bridges to reach another region). Thus, in this study, we regard these two regions as two independent walkable spaces and conduct experiments separately on each of these two regions. On the other hand, the sports events on different days have different schedules and different estimated numbers of audiences, while there is sufficient time to shift temporary stations and supplies at night. Therefore, in this study, we build different models for different days to make the application scenario more practical and to reduce the computation time for each model. In total, there will be 32 models (a combination of 16 days and 2 walkable spaces) to solve the problem in different contextual environments and people flows with a single group of parameters.

The optimization including the genetic algorithm and the proposed model is implemented by C +  + in the software called Qt in Ubuntu Linux 14.04.2. There are 2 CPUs where each of which is Intel (R) Xeon (R) CPU E5-2699 v3 @2.30 GHz. For the parameters of GA, the generation is set as 2000, the population size is set as 3000, while the rates of selection, crossover and mutation are 0.4, 0.8 and 0.3, respectively.

#### Result statistic and visualization

**Fig. 2:**
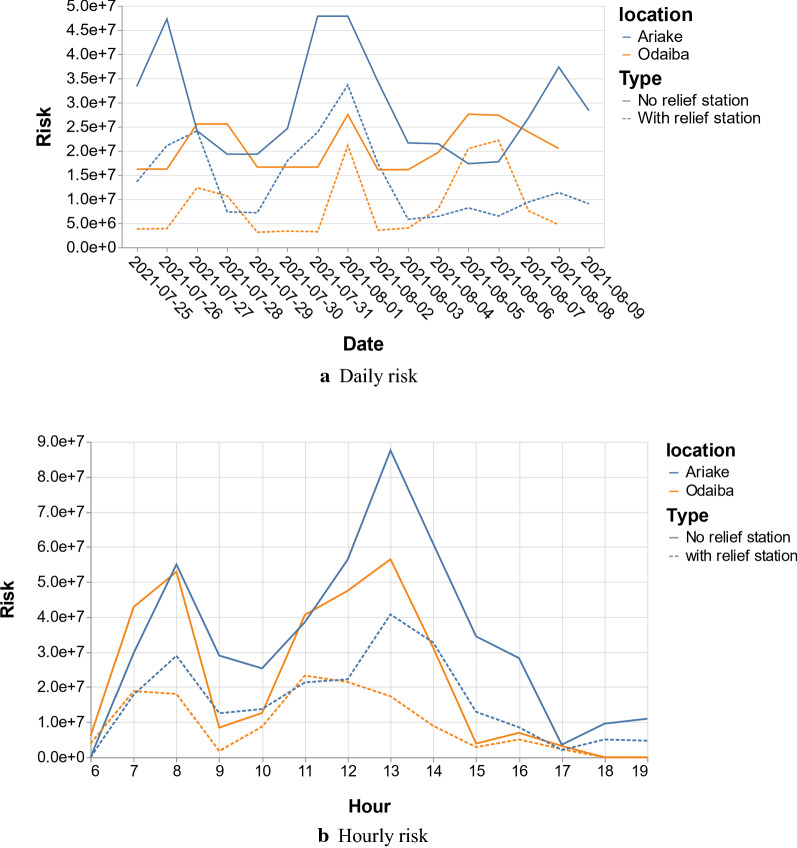
Risk in Odaiba and Ariake area during Olympic games

The results and the visualizations use the solution with a group of parameters in which the station number is 10, the total supply volume is 100 and the maximum supply volume in each station is set to 20. The two plots in Fig. 2 respectively show the optimized total risk as well as the expected risk for the shortest paths without relief stations in different days and hours in different areas during the Olympic games. In particular, the solid lines denote the expected heatstroke at different days and hours without any relief stations, while the dashed lines denote the heatstroke risk with relief stations.

From the plots, we can find that the risk varies in different days and hours with several peaks in several days and hours. Daily and hourly differences indicate that heatstroke risk is very sensitive to the Olympic schedule. Additionally, hourly differences can also reflect weather variation within a day. This is noticeable in the daily risk change, besides the peak observed at 1 p.m., due to both the busy schedule of events and high temperature. The observed peak at 8 a.m. is mainly due to the game schedule. This result mainly could contribute to the efforts made by the Tokyo government aiming at reducing the heatstroke risk for outdoor sports events. Although the performance varies at different hours and days, correctly setting relief stations and optimizing routes can significantly reduce total risk in different event scenarios.

**Fig. 3:**
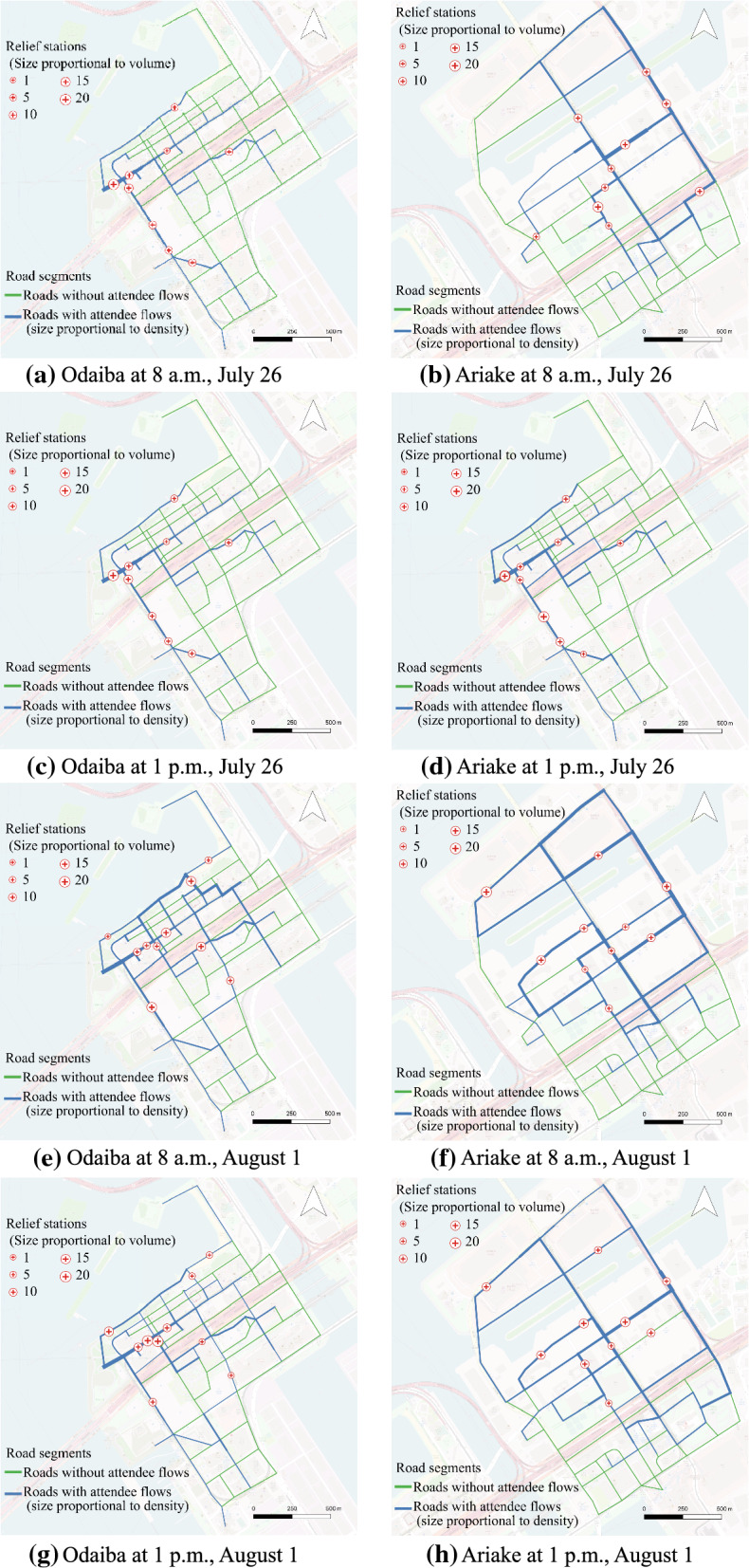
Visualization of event flow density, relief station location and supply volume in each station

Since there are too many events to be visualized in maps, we selectively visualize the flow density, optimized facility location and the supply volume of each facility in both Odaiba and Ariake area at 8 a.m. and 1 p.m. on July 26 and August 1 respectively in 8 maps of Fig. 3. From these maps, we can observe that the supply volume and pedestrian flow density vary at different hours in a day, which stresses the significance of optimizing supply volume within one day. On the other hand, the changes in the optimized locations and flow density at different days suggest the necessity of setting different relief stations on different days.

#### Sensitivity analysis

**Fig. 4:**
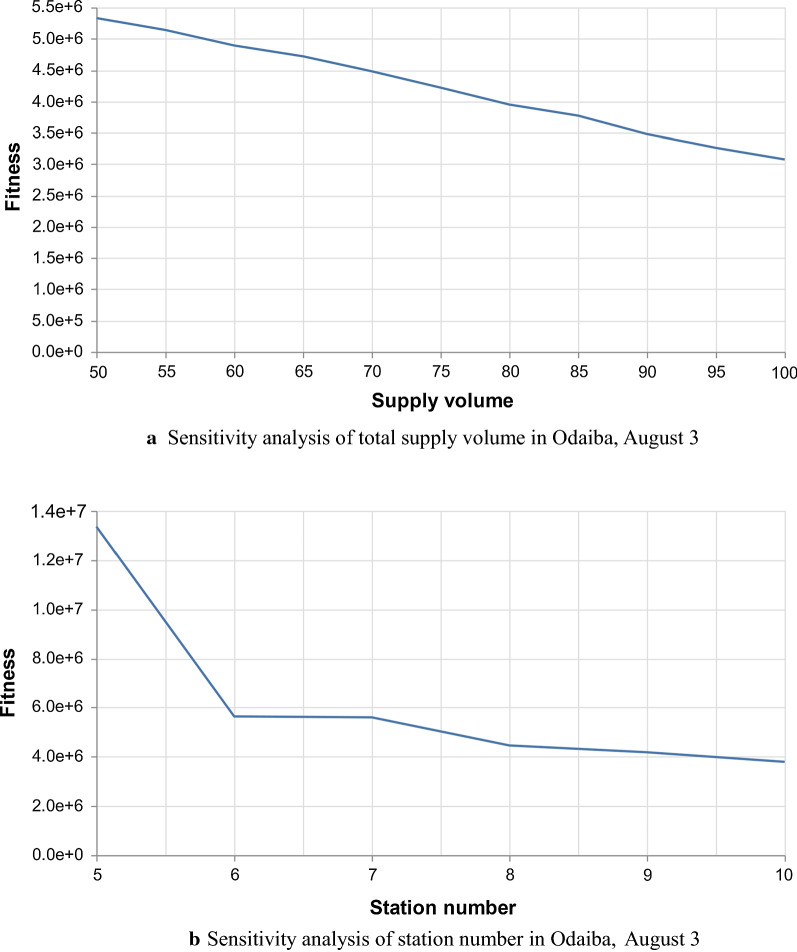
Sensitivity analysis: fitness functions in relation to station numbers and supply volumes

We now conduct sensitivity analysis on the supply volume and stations. Figure 4 shows the sensitivity results for the data of Odaiba on the 3rd, August.

The increase in the total supply volumes or the number of relief stations will reduce the value of the fitness function. This means that increasing the number of supplies (e.g., the number of volunteers or the number of relief resources such as bottled water) or relief stations will either reduce the total risk or reduce the probability that the variables will not meet the constraints in the presented model.

From the results shown in Fig. 4a, the fitness basically decreases linearly with the increase in the number of supply volumes. This shows that in the experiments, 100 units of supply volume may not have reached the upper limit to minimize the total risk in Odaiba area on 3rd, August. However, for the number of relief stations, as shown in Fig. 4b, the rate of fitness reduction gradually decreases along with the increase in the number of stations and tends to remain stable.

On the other hand, when the number of supply volume and the number of relief stations are greater than 50 and 5, respectively, for each flow, the risk between adjacent relief stations on its path has already met the constraint of being less than the maximum risk. Therefore, these solutions are feasible. Overall, when resources are limited, such as the maximum supply volume is 100 and the maximum number of relief stations is 10, the more supply volumes and relief stations, the lower the total risk value is.

#### Ablation study

We conduct several ablation analyses to directly evaluate the performance of our method. Specifically, we compare the fitness between our model and other ablated model settings which are listed as follows:

1. Fixed routes: with the optimized station location, we set all routes of each flow as the shortest routes.
2. Fixed volume: with the optimized station location and routes, we set fixed and equal supply volume, i.e., 10 volume units in each station when the total volume is 100 and the station number is 10.
3. No station: only routes are optimized for each OD and no relief stations are set.

The ablation analysis results conducted on different dates and areas are given in Table 1. In the table, the bold values are the best solutions. Generally, we can observe an increase of the fitness value under different tested assumptions, which results from the poor performance of stations and routes under the risk model. Also, we can observe some large difference between the ablated results and our models, which results from the large penalty value generated in the ablated models.

**Table 1:** Ablation analysis based on fitness

	Odaiba				Ariake			
Date 2021	Our method	Fixed routes	Fixed volume	No station	Our method	Fixed routes	Fixed volume	No station
07–25	**3.83E + 06**	3.99E + 06	3.87E + 06	1.62E + 07	**1.36E + 07**	**1.36E + 07**	1.39E + 07	3.33E + 07
07–26	**3.90E + 06**	4.31E + 06	3.96E + 06	1.62E + 07	**3.11E + 07**	**3.11E + 07**	4.11E + 07	7.73E + 07
07–27	**3.24E + 07**	3.27E + 07	7.27E + 07	4.56E + 07	**5.41E + 07**	5.82E + 07	5.43E + 07	6.42E + 07
07–28	**4.07E + 07**	4.08E + 07	4.08E + 07	6.56E + 07	**7.37E + 06**	1.71E + 07	7.49E + 06	3.93E + 07
07–29	**3.15E + 06**	3.61E + 06	3.26E + 06	1.66E + 07	**7.18E + 06**	7.24E + 06	7.29E + 06	2.93E + 07
07–30	**3.38E + 06**	2.35E + 07	3.53E + 06	3.66E + 07	**5.80E + 07**	6.68E + 07	**5.80E + 07**	7.46E + 07
07–31	**3.26E + 06**	3.40E + 06	1.33E + 07	3.66E + 07	**4.39E + 07**	5.37E + 07	4.40E + 07	7.79E + 07
08–01	**3.11E + 07**	3.15E + 07	3.12E + 07	5.75E + 07	**8.36E + 07**	9.62E + 07	8.38E + 07	1.18E + 08
08–02	**3.57E + 06**	3.76E + 06	3.67E + 06	5.61E + 07	**2.73E + 07**	5.62E + 07	2.74E + 07	7.44E + 07
08–03	**4.01E + 06**	4.26E + 06	4.10E + 06	3.61E + 07	**5.84E + 06**	5.88E + 06	5.94E + 06	2.17E + 07
08–04	**1.80E + 07**	3.80E + 07	1.81E + 07	4.97E + 07	**6.42E + 06**	**6.42E + 06**	6.53E + 06	2.15E + 07
08–05	**5.05E + 07**	6.06E + 07	**5.05E + 07**	7.76E + 07	**8.19E + 06**	2.81E + 07	8.23E + 06	3.73E + 07
08–06	**4.22E + 07**	6.28E + 07	4.23E + 07	6.74E + 07	**2.65E + 07**	3.65E + 07	**2.65E + 07**	4.77E + 07
08–07	**1.76E + 07**	2.76E + 07	1.77E + 07	4.39E + 07	**9.40E + 06**	9.57E + 06	9.68E + 06	4.69E + 07
08–08	**4.72E + 06**	4.78E + 06	4.99E + 06	2.05E + 07	**1.14E + 07**	2.14E + 07	**1.14E + 07**	5.73E + 07
08–09					**9.04E + 06**	1.90E + 07	9.17E + 06	4.83E + 07

## Discussion

### Results discussion

From the results reported in “Result statistic and visualization” section, the Olympic schedule, solar radiation and the relief station have obvious effects on the total risk. A busy schedule of Olympic events and strong solar radiation (high temperature) will increase the total risk, as shown in Fig. 2. On the contrary, the setting of rescue stations can effectively reduce the risk, as shown in Fig. 2 and Table 1. These results are consistent with the description of Eq. (3). The Olympic schedule determines the flow density on the road. Risk is proportional to the flow density and solar radiation, while it is inversely proportional to the number of relief stations and the supply volumes. Therefore, for the Olympic Games' organizers, planning a schedule reasonably and setting up relief stations would be the key ways to reduce the total risk, which also reflects the necessity of optimizing the layout of relief stations and the supply volumes scheduling in this research.

The sensitivity analysis illustrates that the risk decreases as relief stations and supply volumes increase. For the experiment in this research, the setting of the number of relief stations (10) is reasonable, while the supply volumes can be further increased. From Fig. 4, it can be found that continuing to increase the supply volumes will further reduce the total risk and the setting of 100 units has not yet reached the upper limit. However, the increase in the supply volume will bring an increase in operational costs. It is thus necessary to balance these two factors in the optimization, however, note that this research does not take operational costs into the consideration. Therefore, future work will improve this limitation and multi-objective optimization between risk and cost needs to be studied.

The ablation study illustrates the advantages of the proposed model which can be used to optimize the relief station layout, supply volume scheduling and recommended routes of pedestrians, simultaneously. Comparing the proposed model and the fixed routes model, almost all the results of the former are better than the latter. This indicates that the route with the shortest distance may not be the route with the least risk, because the addition of relief stations is another reason for the risk reduction. Therefore, it is essential to optimize relief stations and routes at the same time. For pedestrians, selecting the recommended routes which have been optimized is a good way to reduce the heatstroke risk. Besides, looking at the results of the model of fixed volume we observe that all its results are not better than the results of the proposed model, proving the effectiveness of the optimization of volume scheduling at different hours of each day. Furthermore, the results of the model with no stations further illustrate the advantage of our proposal. The addition of relief stations can significantly reduce the total risk, and its impact is greater than the optimization of routes and volume schedule.

### Method discussion

The advantages of the proposed method are that it does not only plan the placement of relief stations and supply volume scheduling but also determines the optimal routes for the dynamic pedestrian flows. Besides, it is a general method that also has social values that are not only confined to Tokyo Olympic Games. With the indicators and metrics for other types of risks, our method is potentially applicable for solving optimization problems in other specific scenarios that require facility and route optimization in large walkable spaces such as large theme parks and big outdoor exhibitions in any other countries with the risks that are also context-sensitive. In addition, although it cannot be applied directly in daily life as we cannot estimate the flow demand in each road and it is not realistic to enforce all passengers to walk in the fixed route, the concept of our heatstroke risk model can still be applied in practice with different parameters, constraints and other optimization contents such as setting vending machines or planting trees to reduce vulnerability.

Nevertheless, this study has the following limitations on data and models. First, the population simulation could not be evaluated in the current stage since there was no such a big event held with flow data and detailed schedules being provided, as well as it is difficult for local governments to replicate our experiment even with the upcoming big events. In addition, although various prior studies support our choice of contextual information for modeling the heatstroke risk, it is still an assumption that the contextual information utilized in our analysis has an actually high influence in a specific scenario that we focus on, while other factors such as topology, different walking speed and heatstroke resistance of pedestrians among different age groups are not included in the current study. With this, the model is then still not perfect enough for representing real-world scenarios. Besides, we use an MINLP model for the optimization with tens of thousands of variables, which suggests that it needs more validation for the cases of larger and more complicated walkable spaces than ones used for the Tokyo Olympics.

In the future study, we will first try to improve the limitations of our work. Specifically, we will simulate pedestrian flows and evaluate contextual information with more observational data and infrastructure data from several datasets. Besides we will propose a better model with the consideration of operational costs that apply improved, more effective algorithms to optimize routes, facilities and SVF-related infrastructures. Finally, with the improved data and methods, we will try to apply our framework to other application scenarios.

## Conclusions

This study proposed a novel emergency service problem that can be applied in large outdoor event scenarios with multiple walking flows and a novel framework to evaluate the heatstroke risk in walkable spaces during large events by utilizing context-aware indicators that are generated by large and heterogeneous data including facilities, road networks and street view images. Based on a heatstroke risk model, we minimize the total heatstroke risk by solving an MINLP problem for optimizing routes of pedestrians, determining the location of relief stations and the supply volume in each relief station. To illustrate the effectiveness of the proposed model, we then conduct a case study on the planned site of the Tokyo Olympics that is simulated during the two weeks' long period of the Olympic schedule. The social value of the proposed framework is that it not only helps provide layout and scheduling of service facilities and volumes for government and event holders but also recommends routes for pedestrians to reduce the heatstroke risk (or other risks) during large-scale outdoor events.

## Data Availability

https://www.wbgt.env.go.jp/en/wbgt.php; https://www.tripadvisor.jp/Attractions-g298184-Activities-a_allAttractions.true-Tokyo_Tokyo_Prefecture_Kanto.html, based on the ranking on August 25, 2020; https://www.wbgt.env.go.jp/wbgt_data.php; https://tokyo2020.org/ja/schedule, in this study we use the schedule before the games' postponement. https://www.shochi-honbu.metro.tokyo.lg.jp/TOKYO2016_15_9.pdf, in this study we use the schedule before the games' postponement.

## Abbreviations

ACO: Ant colony optimization
AED: Automated external defibrillator
EFLO: Emergency facility location optimization
GA: Genetic algorithm
LBS: Location-based services
LBSN: Location-based social networking
MCLP: Problem to maximize covering location
MEXCLP: Maximum expected coverage location problem
MINLP: Mixed integer nonlinear programming
OD: Origins and destinations
OPAD: Off-site public access devices
OSM: OpenStreetMap
POI: Points of interest
SVF: Sky view factor
TWC: Tokyo Waterfront City
UGCoP: Uncertain geographic context problem
WBGTr: Wet bulb globe temperature

## Indices and sets

*E*: The ID set of all edges in the road network, each one denoted by indices *i* and *j* indicating start and end node of an edge
*E*^*EE*^: The set of the edges connected with end node of each flow at all time intervals, each denoted by indices *i* and *j*,$E^{EE} \in E$
*E*^*FP*^: The ID set of selected edges of the flow path in the road network, denoted by indices *i* and *j*, $E^{FP} \in E$
*E*^*SE*^: The ID set of the edges connected with start node of each flow at all time intervals, each denoted by indices *i* and *j*,$E^{SE} \in E$
*F*: The ID set of flows, denoted by the index *f*
*S*: The ID set of stations, denoted by* s* and *k*
*T*: The ID set of candidate time intervals, denoted by the index *t*
*V*: The ID set of all nodes in the road network, denoted by indices $\alpha$ and $\beta$

## Input parameters

*c*: Factor value that increases vulnerability in the road segment, the average SVF value in this paper
*L*_*i*_: Road length (or travel time) of edge *i*
*N*^*T*^: The number of time intervals
$N_{t}^{F}$: The number of flows at time interval *t*
$N_{f,t}^{P}$: The number of people of flow *f* during time interval *t*
*N*^*V,max*^: The max supply volumes of all stations
*N*^*SV,max*^: The max supply volumes of one station
*N*^*S,max*^: The max station numbers
$N_{i,f,t}^{EF}$: The first node ID of the selected edge *i* on flow path *f* at a time interval *t*
$N_{i,f,t}^{ES}$: The second node ID of the selected edge *i* on flow path *f* at a time interval *t*
*RF*^*max*^: The threshold of the maximum risk of edges of flow *f*
*Ri*^*max*^: The threshold of the maximum risk of each edge
*Rs*^*max*^: The threshold of the maximum risk between two adjacent stations on the path of flow *f*
*W*_*t*_: Hazard factor denoted by WBGT score during a time interval

## Decision variables

$B_{i,f,t}^{P}$: Binary variable, 1 if a flow *f* is observed on edge *i* at a time interval *t*, 0 otherwise
$E_{s}^{S}$: Integer variable refers to the ID of the edge which has a station *s*
$N_{s,t}^{V}$: Integer variable refers to the volume of service station *s* at a time interval *t*

## Intermediate variables

$a_{i,j,f,t}$: The value of each element in the adjacency matrix of selected edges for flow *f* at time interval *t*
$B_{i}^{S}$: Binary variable, 1 if there are some stations are established on edge *i*; 0 otherwise
$N_{i,t}^{V}$: Integer variable refers to the volume of service in a station on edge *i* at a time interval *t*
$n_{f,t}^{E}$: The number of edges on flow path *f* at time interval *t*
$p_{i,j,f,t}$: The value of each element in the accessibility matrix of selected edges for flow *f* at time interval *t*
$R_{i,f,t}$: Refers to the risk value of edge *i* of flow *f* at a time interval *t*
$R_{i,t}^{E}$: Refers to vulnerability on edge *i* at a time interval *t*
$R_{i,t}^{V}$: Refers to exposure on edge *i* at a time interval *t*
$V_{i,t}^{R}$: Factor value that decreases vulnerability in the road segment *i* at time interval *t*

## Footnotes

1 https://www.wbgt.env.go.jp/en/wbgt.php.

2 https://www.tripadvisor.jp/Attractions-g298184-Activities-a_allAttractions.true-Tokyo_Tokyo_Prefecture_Kanto.html, based on the ranking on August 25, 2020.

3 https://www.wbgt.env.go.jp/wbgt_data.php.

4 https://tokyo2020.org/ja/schedule, in this study we use the schedule before the games' postponement.

5 https://www.shochi-honbu.metro.tokyo.lg.jp/TOKYO2016_15_9.pdf, in this study we use the schedule before the games' postponement.
